# Efficacy and safety of Shenqi Yangxin formula in patients with stable coronary artery disease based on CCTA: rationale, design, and study protocol for a randomized, double-blind, placebo-controlled study

**DOI:** 10.3389/fcvm.2026.1697750

**Published:** 2026-05-08

**Authors:** Xinyu Zhang, Yicheng Liu, Jinghui Sun, Yunru Chen, Xiao Zhang, Jiaji Guo, Hua Ren, Peng Liu, Chenglong Wang, Dawu Zhang

**Affiliations:** 1 National Clinical Research Center for Chinese Medicine Cardiology, Xiyuan Hospital, China Academy of Chinese Medical Sciences, Beijing, China; 2 Department of Cardiovascular, Xiyuan Hospital, China Academy of Chinese Medical Sciences, Beijing, China; 3 State Key Laboratory of Traditional Chinese Medicine Syndrome, Xiyuan Hospital, China Academy of Chinese Medical Sciences, Beijing, China

**Keywords:** CCTA, Chinese herbal medicine, protocol, randomized controlled trial, stable coronary artery disease

## Abstract

**Background:**

Among patients with stable coronary artery disease (SCAD), those with moderate coronary artery stenosis remain at high risk for adverse cardiovascular events despite optimal medical therapy. Reducing residual cardiovascular risk, particularly inflammation-related risk, is a key target for improving long-term outcomes. Evaluation of plaque burden and hemodynamics in patients with SCAD based on coronary computed tomography angiography (CCTA)-derived techniques, along with risk stratification and prediction, has become a hotspot, holding significant importance for standardizing clinical management of SCAD. Shenqi Yangxin Formula (SYF), a traditional Chinese medicine compound, has been widely used in China for SCAD and shown to alleviate symptoms, but its mechanistic benefits remain unclear. Based on this, we proposed that SYF could benefit patients with SCAD (qi deficiency and blood stasis syndrome) by reducing residual cardiovascular risk and improving coronary hemodynamics. This clinical trial was designed to objectively evaluate the efficacy and safety of SYF in SCAD.

**Methods:**

This is a randomized, double-blind, placebo-controlled trial, enrolling 60 SCAD patients meeting criteria for qi deficiency and blood stasis syndrome. Participants are randomized 1:1 to receive SYF or placebo in addition to guideline-directed medical therapy for 12 weeks at a loading dose, followed by 12 weeks at half dose.

**Results:**

As of July 2024, enrollment is complete, with trial completion expected in the first quarter of 2026. The primary outcomes are changes in CCTA-derived coronary hemodynamic parameters (CT-derived fractional flow reserve, wall shear stress) and fat attenuation index at 24 weeks. Secondary outcomes include imaging characteristics of plaques, traditional chinese medicine syndrome scores, seattle angina questionnaire scores, echocardiography, adverse cardiovascular events, and metabolomic profiles. Participants undergo follow-up at 4, 12 and 24 weeks post-randomization. All data will be collected in case report forms and analyzed according to predefined statistical plans.

**Conclusion:**

This study will provide novel evidence on the efficacy and safety of SYF in improving coronary hemodynamics and reducing residual inflammatory risk in patients with SCAD and moderate stenosis, potentially offering a complementary therapeutic strategy.

**Clinical Trial Registration:**

http://123.57.9.4:8085/zh-CN/Home/ProjectView?pid=dbc777bb-434e-44a4-af75-cfabc32d64db, identifier ITMCTR2024000215.

## 1 Introduction

Driven by metabolic risk factors, the global burden of cardiovascular disease—primarily due to the rising prevalence of atherosclerosis (AS)—is expected to increase in the coming decades (1). Coronary artery disease (CAD) represents the predominant clinical manifestation of AS. Despite adherence to guideline-directed medical therapy (GDMT), patients with stable coronary artery disease (SCAD), particularly those classified as CAD-RADS category 3 (moderate stenosis, 50%–69%), continue to face an elevated risk of adverse cardiovascular events (2–4). Large-scale randomized trials and cohort studies such as SCOT-HEART, ISCHEMIA, and PROMISE have shown that routine invasive revascularization does not reduce mortality or myocardial infarction rates in this population (5). Increasing evidence suggests that plaque morphology and burden—especially the presence of high-risk plaque features—are more robust predictors of adverse events (6–8). Consequently, the therapeutic paradigm for SCAD has shifted from solely relieving ischemia toward promoting plaque stabilization (9).

Statins remain the cornerstone therapy for lowering low-density lipoprotein cholesterol (LDL-C) in SCAD; however, many patients continue to exhibit substantial residual metabolic, inflammatory, and thrombotic risks (10). Among these, chronic vascular inflammation has emerged as a key driver of residual cardiovascular risk (11). While anti-inflammatory agents, such as low-dose colchicine, have shown encouraging results (12, 13), recent trials have reported inconsistent outcomes and raised concerns regarding long-term tolerability (14). Hence, there is a pressing need for effective therapeutic strategies aimed at mitigating residual risk and promoting plaque stabilization or regression.

Coronary computed tomography angiography (CCTA), as a first-line, non-invasive alternative to coronary angiography, is now widely applied in the diagnosis and management of SCAD (15). Advances in CCTA-derived techniques allow comprehensive assessment of plaque burden, vascular inflammation, and hemodynamic alterations, providing valuable tools for risk stratification and prognostication (16). Numerous studies have identified low-attenuation plaques with lipid-rich necrotic cores, fat attenuation index (FAI), computed tomography-derived fractional flow reserve (CT-FFR), and regions of low wall shear stress (WSS) as independent predictors of adverse cardiovascular outcomes (8, 17–19). In particular, FAI has demonstrated considerable potential for evaluating residual inflammatory risk (17), and the presence of high-risk plaques has been associated with a 70% increase in adverse event rates (7). Growing evidence suggests that CCTA can improve risk stratification and guide the timely initiation and intensification of preventive therapies, including statins and antiplatelet agents, thereby reducing the risk of adverse events such as cardiac death or myocardial infarction in patients with SCAD and improving prognosis (20–22). In addition, CCTA helps avoid unnecessary or excessive revascularization (23–25), highlighting its important role in promoting standardized clinical management of SCAD (26).

Traditional Chinese medicine (TCM) provides a distinctive therapeutic strategy for SCAD. Previous studies have suggested that TCM interventions may offer advantages in alleviating symptoms, improving quality of life, and reducing the risk of adverse cardiovascular events in patients with SCAD (27, 28). In addition, meta-analyses have indicated that TCM treatment is not inferior to invasive treatment strategies in terms of reducing the incidence of major adverse cardiovascular events (MACE) (29). However, most existing studies have primarily relied on MACE incidence or clinical scores as outcome measures, and objective imaging evidence supporting potential changes in coronary structure and function following TCM intervention remains insufficient. In particular, evidence regarding coronary hemodynamic parameters and residual inflammatory risk assessment is still limited. Therefore, a rigorously designed randomized, double-blind, placebo-controlled trial incorporating advanced imaging techniques is warranted to systematically evaluate the effects of TCM on coronary pathophysiology and residual cardiovascular risk.

In TCM theory, SCAD is categorized under “heart pain”, a classical disease entity characterized by precordial pain analogous to angina pectoris in modern medicine, primarily attributed to “qi deficiency” and “blood stasis”. “Qi deficiency” is a fundamental concept in TCM referring to insufficient vital energy that impairs organ function. In patients with SCAD, qi deficiency commonly manifests as fatigue, shortness of breath, and palpitations, particularly exacerbated by physical activity. “Blood stasis” refers to impaired blood circulation resulting in stagnation and accumulation, which corresponds to the hypercoagulable state in modern medicine characterized by enhanced blood coagulation and weakened anticoagulant function. In the pathogenesis of SCAD, qi deficiency serves as the fundamental pathological basis, while blood stasis represents the resulting pathological product. Qi deficiency leads to insufficient heart qi and impaired blood circulation, thereby promoting the formation of blood stasis and atherosclerotic plaques. “Tonifying qi” refers to the therapeutic principle of supplementing and enhancing the body's vital energy through herbal medicine and other TCM interventions, aiming to restore organ function and strengthen the heart's ability to promote blood circulation. “Activating blood circulation” represents the therapeutic strategy of promoting blood flow, removing stagnation, and improving microcirculation through blood-activating and stasis-resolving agents. In the treatment of SCAD with qi deficiency and blood stasis syndrome, the combination of tonifying qi and activating blood circulation addresses both the root cause and the manifestation, thereby restoring normal hemodynamics and preventing atherosclerotic plaque progression.

Shenqi Yangxin Formula (SYF) is a widely used TCM compound for SCAD, formulated on the principle of “tonifying qi and activating blood circulation”. We previously identified the active components of SYF using ultra-performance liquid chromatography coupled with quadrupole time-of-flight mass spectrometry (UPLC-Q-TOF-MS), including *Chikusetsusaponin Iva*, *Chuanxiongol*, *ginsenoside Rg4*, *ginsenoside Rg5*, *notoginsenoside R2*, *notoginsenoside T5*, *octahydrocurcumin*, *senkyunolide B*, *senkyunolide D*, *vanillic acid*, *verbascoside*, *and 25R inokosterone* (Supplementary Files 1 and 2). Modern pharmacological studies suggest that these components exert multiple effects, including lipid lowering, inhibition of pathological cell proliferation within plaques, attenuation of oxidative stress and ferroptosis, and modulation of inflammatory responses, thereby contributing to plaque stabilization and improvement of SCAD (30–32). Our preliminary clinical practice has also confirmed the clinical efficacy of qi-tonifying and blood-activating Chinese herbal medicines in treating CAD (33).

To date, only limited studies have employed CCTA-derived parameters to evaluate the efficacy of TCM in SCAD. Notably, Professor Wang Jie's team at Guang'anmen Hospital, China Academy of Chinese Medical Sciences (34), and Professor Xu Danping's team from the Eighth Affiliated Hospital of Sun Yat-sen University (35), have investigated TCM interventions on plaque characteristics and provided preliminary evidence for the potential plaque-stabilizing properties of TCM (36, 37). Our study focuses on coronary artery hemodynamic indices and FAI, which have not been previously reported in clinical research on TCM. We hypothesize that SYF improves coronary artery hemodynamic function and reduces residual inflammatory risk, thereby offering a novel and promising therapeutic strategy for patients with SCAD and moderate coronary artery stenosis. Accordingly, we designed this randomized, double-blind, placebo-controlled trial to objectively evaluate the efficacy and safety of SYF in this population using comprehensive CCTA-based assessment.

## 2 Methods

### 2.1 Study design

This study is a randomized, double-blind, placebo-controlled superiority trial. It has been registered on the International Traditional Medicine Clinical Trial Registry Platform (registration number: ITMCTR2024000215) and approved by the Research Ethics Committee of Xiyuan Hospital, China Academy of Chinese Medical Sciences (approval number: 2024XLA060-2), confirming compliance with the Declaration of Helsinki, the 2020 Good Clinical Practice (GCP) guidelines for drug clinical trials, and the International Ethical Guidelines for Biomedical Research Involving Human Subjects (CIOMS). The decision to modify the protocol must be communicated by the Research Ethics Committee of Xiyuan Hospital, China Academy of Chinese Medical Sciences. The design of this study protocol follows the SPIRIT 2013 Statement (38, 39). The completed SPIRIT checklist is available in Supplementary File 3.

**Figure 1:**
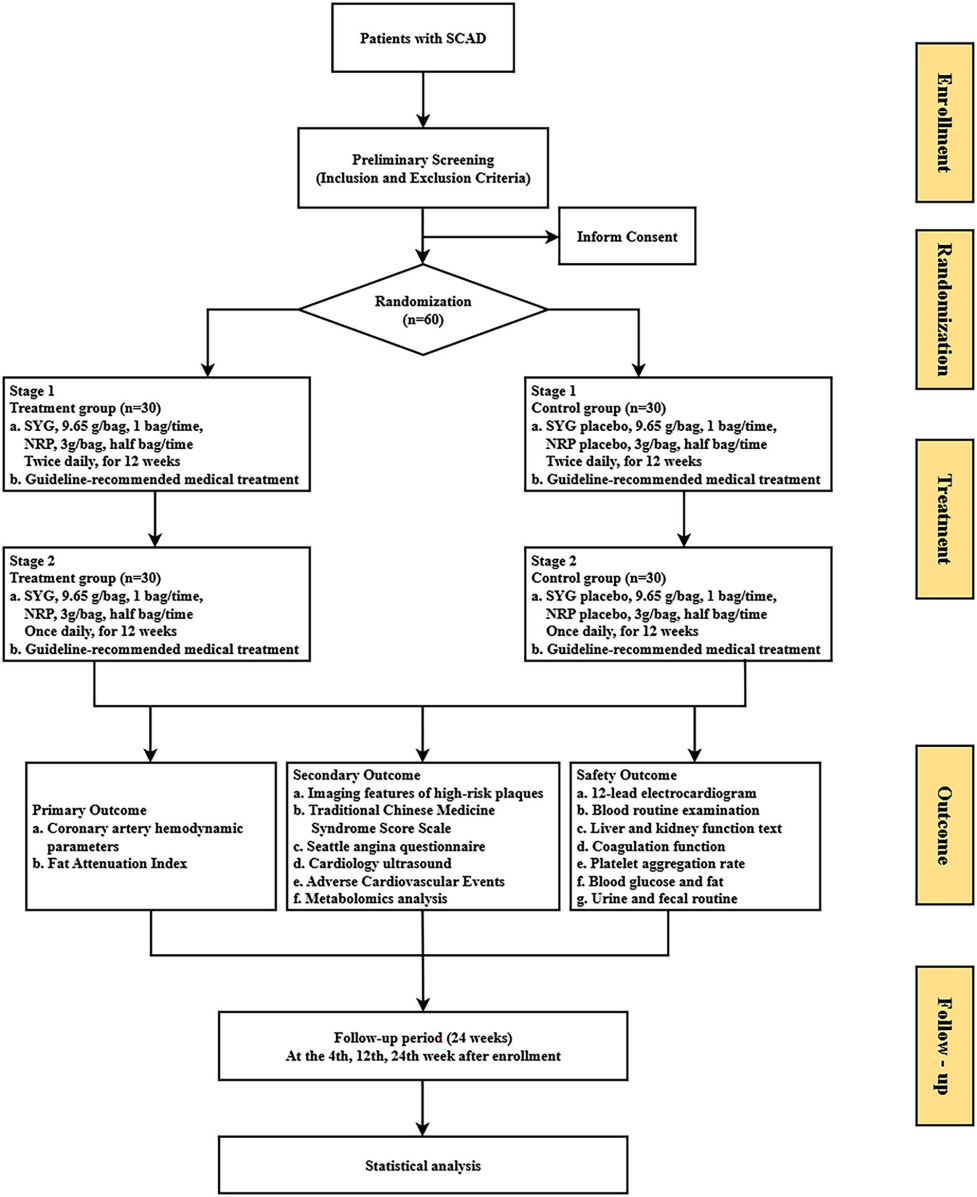
Flow chart of the study design. SCAD, stable coronary artery disease; SYG, Shenqi Yangxin Granule; NRP, Notoginseng Radix powder; GDMT, Guideline-Directed Medical Therapy; coronary artery hemodynamic parameters, include CT-FFR and WSS; TCMSS, traditional Chinese medicine syndrome scale.

This study will be conducted at Xiyuan Hospital, China Academy of Chinese Medical Sciences, with a total of 60 participants to be recruited. After providing written informed consent, participants will be enrolled in the trial, which has a total treatment duration of 24 weeks, with follow-up visits scheduled at weeks 4, 12 and 24 of the intervention (Figure 1 and Table 1).

**Table 1:** Schedule for data collection of this study.

Items	Trial period				
	Enrollment		Post-randomization		Close-out
Timepoint	−7 d to 0d	0d	4w ± 3d	12w ± 3d	24w ± 3d
Enrollment					
Inclusion/exclusion criteria	×				
Informed consent	×				
Basic information	×				
Physical examination	×				
Medical History	×				
Randomization		×			
Intervention					
Treatment group (*n* = 30)		×	→	→	×
Control group (*n* = 30)		×	→	→	×
Assessments					
Coronary artery hemodynamic parameters		×			×
Fat attenuation index		×			×
Imaging features of high-risk plaques		×			×
TCMSS		×	×	×	×
Seattle angina questionnaire		×	×	×	×
Cardiology ultrasound		×			×
Adverse Cardiovascular Events		×	→	→	×
Metabolomics analysis		×			×
12-lead electrocardiogram		×	×	×	×
Blood routine examination		×	×	×	×
Liver and kidney function text		×	×	×	×
Coagulation function		×	×	×	×
Platelet aggregation rate		×	×	×	×
Blood glucose and fat		×	×	×	×
Urine and fecal routine		×	×	×	×

×, The moment of enrollment, intervention or assessments; Right arrow: indicates continued intervention or ongoing monitoring of adverse cardiovascular events. Coronary artery hemodynamic parameters, including CT-FFR and WSS; TCMSS, traditional Chinese medicine syndrome scale.

### 2.2 Participants

#### 2.2.1 Recruitment

Participants will be recruited from the outpatient and inpatient departments of the Cardiovascular Department at Xiyuan Hospital, China Academy of Chinese Medical Sciences. Registration commenced in August 2024. As of July 2025, the study has enrolled 100% of participants and is expected to finish in the first quarter of 2026.

#### 2.2.2 Diagnosis criteria

The diagnostic criteria of SCAD refer to the 2023 AHA/ACC/ACCP/ASPC/NLA/PCNA guideline for the management of patients with chronic coronary disease (40). The diagnosis of Qi Deficiency and Blood Stasis Syndrome is based on the “Expert Consensus on TCM Diagnosis and Treatment of Stable Angina Pectoris of Coronary Heart Disease (2018 Edition)”, the “Guidelines for Clinical Research on New Chinese Drugs”, the “Practical Diagnostic Criteria for Blood Stasis Syndrome”, and Xiyuan Hospital, China Academy of Chinese Medical Sciences' established diagnostic standards for qi deficiency and blood stasis syndrome in CAD (41–42). The diagnostic criteria are as follows:

(a) Primary symptoms: Stabbing or dull ache in the chest;
(b) Secondary symptoms: Fatigue, mental fatigue, palpitations, shortness of breath, spontaneous sweating, and dark purple facial complexion;
(c) Tongue and pulse characteristics: Pale purple tongue with ecchymotic spots, weak and rough pulse.

Patients meeting the primary symptom plus any two or more secondary symptoms, combined with observation of tongue appearance and pulse characteristics, can be diagnosed with qi deficiency and blood stasis syndrome.

#### 2.2.3 Inclusion criteria

(a) CCTA demonstrates at least one major branch with luminal diameter stenosis of 50%–70%, exhibiting soft plaque or mixed plaque types;
(b) Based on CCTA analysis within the past month, CT-FFR >0.8;
(c) Canadian Cardiovascular Society classification of angina pectoris is grade I–II;
(d) Meets the diagnostic criteria for Qi deficiency and blood stasis syndrome;
(e) Aged 30–75 years, with no gender restrictions;
(f) Voluntarily consents to participate and signs the informed consent form.

#### 2.2.4 Exclusion criteria

(a) Acute myocardial infarction or coronary revascularization treatment within the past 30 days;
(b) Presence of any of the following conditions: left main coronary artery stenosis ≥50% or chronic total occlusion; drug-resistant hypertension (systolic blood pressure ≥160 mmHg, diastolic blood pressure ≥100 mmHg); severe malignant arrhythmia, cor pulmonale, rheumatic heart disease, myocarditis, cardiomyopathy, aortic dissection, pulmonary embolism, or other serious diseases;
(c) Patients who have undergone coronary artery bypass graft surgery;
(d) Patients with stents in both the left and right coronary arteries;
(e) Patients presenting with chest pain symptoms related to cervical spondylosis, biliary heart syndrome, esophageal hiatal hernia, neurasthenia, menopausal syndrome, or other related conditions;
(f) Patients with congenital coronary artery anomalies or connective tissue disease with coronary artery involvement;
(g) Individuals at high risk of severe bleeding;
(h) Patients with severe liver disease, or alanine aminotransferase (ALT) and aspartate aminotransferase (AST) levels exceeding three times the upper limit of normal, or those with renal impairment, defined as creatinine clearance <60 mL/min;
(i) Patients with severe hematological disorders or malignant tumors;
(j) Individuals with any of the following contraindications to coronary CTA: ① Patients with contraindications to Ioversol injection (e.g., a history of severe hypersensitivity reactions, severe thyroid disease, renal insufficiency, or any other conditions deemed inappropriate for contrast administration by the investigators). ② Inability to cooperate with scanning and breath-holding. ③ Pregnant or lactating women, or women of childbearing potential planning pregnancy. ④ Clinically unstable vital signs (such as decompensated heart failure, severe hypotension, etc.);
(k) Patients with psychiatric disorders or cognitive impairment;
(l) Patients allergic to the investigational drug;
(m) Participation in other clinical trials within the past month, or oral administration of other Chinese herbal preparations with blood-activating and stasis-resolving effects;
(n) Patients whose CCTA images are unavailable or of insufficient quality to permit plaque characterization or hemodynamic analysis.

### 2.3 Randomization and allocation concealment

All patients who consent to participate in the study and meet the inclusion and exclusion criteria will be randomized in a 1:1 ratio to the control group or the experimental group. The statistical expert from the Clinical Pharmacology Research Institute of Xiyuan Hospital, China Academy of Chinese Medical Sciences, generated the sequence using SAS software (version 9.4) employing a stratified block randomization method, sequentially numbered from 1 to 60. The statistical expert will send the blind codes directly to the drug manufacturing department to label the investigational products. According to the clinical research protocol, the drug randomization code serves as the unique identifier for participants, and treatment allocation will remain undisclosed until the completion of data statistical analysis. Confidential information, including the selected block length, random seed parameters, the randomization sequence list, and emergency envelopes, is sealed in opaque envelopes and maintained by independent personnel from the Clinical Pharmacology Research Institute.

### 2.4 Blinding

Throughout the trial, the investigational drug and placebo are matched in appearance, smell, taste, packaging, and administration regimen. Therefore, participants are unable to identify their group allocation based on the characteristics of the study medication. Investigators and data collection staff distribute the study medication according to the participant's randomization ID only, and they do not have access to the randomization sequence or allocation information described in Section 2.3. For blinded outcome assessment, CCTA image analysis is performed by senior radiologists from the Department of Radiology, Xiyuan Hospital, China Academy of Chinese Medical Sciences, who are independent of the trial implementation team. The image assessors, blinded to both treatment allocation and follow-up time points, evaluate CT-FFR, WSS, FAI, and plaque characteristics according to a pre-specified standardized analysis workflow, thereby minimizing assessment bias as much as possible. After data collection and entry into the electronic database are completed, independent personnel from the Institute of Clinical Pharmacology, Xiyuan Hospital, China Academy of Chinese Medical Sciences, code and securely store the allocation information using “Group A/Group B”. The statistician receives only the coded dataset for analysis and remains blinded to the actual intervention assigned to each group. The decoding list is unblinded only after completion of the primary statistical analysis. In the event of a serious adverse event requiring urgent clinical management, individual emergency unblinding is performed according to the predefined procedure, and the reason and time of unblinding are documented.

### 2.5 Interventions

GDMT includes the use of antiplatelet agents, β-blockers, and renin-angiotensin-aldosterone system inhibitors, etc. (40). Based on the patient's clinical condition, the use of Hypoglycemic drugs, antihypertensive drugs, lipid-lowering drugs, and other pharmacological treatments is permitted. Investigators should accurately document concomitant medications and maintain dosage stability throughout the trial period.

#### 2.5.1 Treatment group

The composition of SYF is shown in Table 2. *Ginseng Radix et Rhizom*, *Chuanxiong Rhizoma*, *Cyathulae Radix, Forsythiae Fructus*, *Curcumae Radix* were procured from Beijing Tcmages Pharmaceutical Co., Ltd. *Ginseng Radix et Rhizom* formula granules' batch number: 23023281, *Chuanxiong Rhizoma* formula granules' batch number: 24008491, *Forsythiae Fructus* formula granules' batch number: 23018951, Cyathulae Radix formula granules’ batch number: 24012561, and *Curcumae Radix* formula granules' batch number: 23015951. The herbs were processed through decoction, filtration, and concentration to produce Shenqi Yangxin granules (SYG), packaged as 9.65 g per bag. *Notoginseng Radix* powder (NRP) was procured from China Pharmaceutical Company, Batch number: 740711102, packaged in 3 g per bag.

**Table 2:** The composition of shenqi yangxin formula (intervention drug).

Chinese name	Scientific name	Latin name	Species name	Weight (%)
Ren shen	Panax ginseng C. A. Mey.	*Ginseng Radix et Rhizoma*	Araliaceae	18.75
Chuan xiong	Ligusticum chuanxiong Hort.	*Chuanxiong Rhizoma*	Umbelliferae	18.75
Niu xi	Cyathula officinalis Kuan	*Cyathulae Radix*	Amaranthaceae	25.00
Lian qiao	Forsythia suspensa (Thunb.) Vahl	*Forsythiae Fructus*	Oleaceae	12.50
Yu jin	Curcuma wenyujin Y. H. Chen et C.Ling	*Curcumae Radix*	Zingiberaceae	18.75
San qi	Panax notoginseng (Burk.)F. H. Chen	*Notoginseng Radix*	Araliaceae	6.25

Stage one: one bag of SYG and half a bag of NRP per dose, twice daily, mix with 100 mL of hot water and take orally. The treatment duration is 12 weeks. Participants are required to return unused medication and packaging to the investigators at each visit to assess medication adherence.

Stage two: one bag of SYG and half a bag of NRP per dose, once daily, mix with 100 mL of hot water and take orally. The treatment duration is 12 weeks. Participants are required to return unused medication and packaging to the investigators.

The dosing regimen of 12 weeks at a full dose followed by 12 weeks at a half dose was designed based on the following three considerations:

(a) Regarding efficacy exploration and traditional usage experience, our team's previous clinical studies have demonstrated that a 12-week treatment course is the conventional duration for Qi-tonifying and blood-activating TCM in treating stable coronary artery disease, and this duration has been shown to achieve certain clinical efficacy (28, 43). This study incorporates an additional 12-week half-dose maintenance phase to explore whether extending the medication duration can provide patients with additional long-term clinical benefits while maintaining overall drug efficacy.
(b) Concerning patient compliance, chronic management of coronary artery disease requires long-term medication adherence, and a 24-week medication period is relatively prolonged. Reducing the medication dose in the latter 12 weeks helps alleviate medication fatigue among patients, thereby improving adherence during this extended clinical trial.
(c) From a health economic perspective, reducing the medication dose in the second half of the trial effectively lowers treatment costs. If this study demonstrates the efficacy of this regimen, it will provide evidence-based support for future clinical practice, enabling clinicians to reduce the medical financial burden on patients and society while ensuring therapeutic efficacy.

#### 2.5.2 Control group

The primary component of the SYG placebo is dextrin, which was procured by Beijing Tcmages Pharmaceutical Co., Ltd, Batch number: 240601202404121, and the primary component of the NRP placebo is medical starch, which was procured by Liaoning Dongyuan Pharmaceutical Co., Ltd., Batch number: F20190001996. Furthermore, 10% of the raw drug was added to the SYG placebo and NRP placebo to match the odor, color, taste, and texture of the experimental group medication. The packaging, administration, and dosage of the placebo are identical to those of the experimental group. Participants are required to return unused medication and packaging to the investigators after each stage.

#### 2.5.3 Removal, dropout, and termination criteria

Participants will be excluded from the study if any of the following conditions are found after the study finishes: a. Participants don't meet the inclusion criteria or meet the exclusion criteria; b. Participants don't adhere to the medication regimen as specified in the study protocol; c. Participants have no post-treatment visit records; d. Participants used concomitant medications during the trial that may affect the evaluation of the efficacy and safety of SYF. Excluded cases should not be included in the efficacy analysis. However, the safety analysis should include those who have received at least one treatment and have at least one safety record.

If participants experience adverse events such as cardiac death, non-fatal myocardial infarction, acute heart failure, severe angina pectoris, severe arrhythmia, or coronary revascularization (PCI or CABG) during the study, or significant deviations occur during the intervention that hinder the evaluation of drug efficacy, participants will be withdrawn from the trial based on the clinical physician's judgment. Participants may voluntarily withdraw from the trial if they are dissatisfied with the efficacy, experience adverse reactions, plan to receive other treatments, or for other reasons leading to unwillingness to continue treatment. Participants who fail to complete the observation period specified in the trial will be considered dropout cases, regardless of the timing or reason. The reasons for dropout will be documented in the case report form (CRF) and included in the final data analysis.

The entire study will be terminated under the following circumstances: a. Occurrence of multiple serious adverse events related to the trial that pose a significant threat to participant safety; b. Quality issues with the investigational drug; c. Unblinding of the entire double-blind trial, or an emergency letter unblinding rate exceeding 20%. The investigator must provide a comprehensive explanation to the ethics committee regarding the reasons for trial termination, submit a written application for termination, and the ethics committee will make the final decision on whether to terminate the trial. If the study is terminated, the investigator shall ensure that participants continue to receive necessary treatment and follow-up after trial termination. Simultaneously, all relevant documents and materials must be properly preserved to assess the trial outcomes and reasons for termination.

#### 2.5.4 Drugs combined and contraindicated in trial

Patients experiencing angina pectoris attacks may take sublingual nitroglycerin or rapid-acting rescue pills. However, the investigator must record the specific date of onset, duration, and relief measures. In addition to the initially administered medications, the investigator shall record all additional drugs and treatments given to the patient in the CRF. During the study period, the use of other traditional Chinese medicine decoctions, intravenous injections, or proprietary Chinese medicines with qi tonification and blood circulation activation effects is prohibited. Acupuncture, cupping, and other appropriate traditional Chinese medicine techniques should not be accepted as treatments.

### 2.6 Outcomes

#### 2.6.1 Primary outcomes

The primary outcome measures of this study are coronary artery hemodynamic parameters and FAI assessed by CCTA. The coronary artery hemodynamic parameters include CT-FFR and WSS. These parameters will be evaluated at baseline and 24 weeks after randomization.

#### 2.6.2 Secondary outcomes

The secondary outcome measures of this study include imaging features of plaques, Traditional Chinese Medicine Syndrome Score (TCMSS), the Seattle Angina Questionnaire (SAQ), echocardiography, adverse cardiovascular events, and metabolomic analysis.

The imaging features of plaques include plaque morphology, location, volume, degree of stenosis, and calcification score, all of which will be assessed at baseline and 24 weeks after randomization. Echocardiography and metabolomics analyses will be performed at baseline and 24 weeks after randomization; TCMSS and SAQ will be recorded at baseline and 4th, 12th, 24th weeks after randomization. Adverse cardiovascular events are defined as cardiac death, non-fatal myocardial infarction, coronary revascularization, stroke, severe angina pectoris, and hospitalization due to acute coronary syndrome, malignant arrhythmia, or heart failure occurring within 24 weeks after randomization, and will be recorded at any time following participant enrollment.

#### 2.6.3 Safety outcomes and adverse events report

Safety will be assessed by monitoring adverse events (AEs), serious adverse events (SAEs), withdrawals, or treatment modifications caused by AEs. Additionally, this study includes safety indicators such as 12-lead electrocardiogram, blood routine examination, liver and kidney function tests, coagulation function, platelet aggregation rate, blood glucose and lipid profiles, as well as urine and fecal routine examinations, which will be performed at baseline and 4th, 12th, 24th weeks following randomized grouping.

#### 2.6.4 CCTA acquisition and image analysis

CCTA examinations were performed using a GE Revolution CT scanner. Scanning parameters included a tube voltage of 120 kV and tube current of 370–450 mA, with automatic exposure control technology applied to adapt to patient body habitus. The scanning mode was selected as either prospective or retrospective ECG-gating based on heart rate, requiring only a single axial scan to complete image acquisition. Images were reconstructed with a slice thickness of 0.625 mm and a rotation time of 0.28 s. A non-ionic contrast agent (Ioversol injection, 350 mg/mL, Jiangsu Hengrui Pharmaceuticals Co., Ltd.) was administered intravenously at a rate of 5 mL/s, with a total volume of 1 mL/kg. Following contrast injection, 30 mL of normal saline was injected at the same rate using the Smart bolus tracking technique, with the descending aorta selected as the region of interest to achieve optimal arterial phase imaging at 80 HU. Images were reconstructed using the ASiR iterative reconstruction algorithm, with SSF2 selected as the optimal reconstruction kernel for subsequent analysis. Image analysis was performed using the dedicated CoronaryDoc V2.0 software [Yukun (Beijing) Network Technology Co., Ltd.]. The specific methods for analyzing the images are as follows:

(a) CT-FFR Assessment: CT-FFR was utilized as the core indicator of vascular function. The CT-FFR value was measured 2 cm distal to the stenotic lesion. For patients with multiple stenoses or multi-vessel involvement, the vessel with the most severe luminal stenosis and its most severe lesion were selected as the measurement point. The change before and after treatment was calculated (△CT-FFR = post-treatment − pre-treatment).
(b) FAI Assessment: FAI was obtained to reflect the local inflammatory burden of the coronary artery. The FAI level was measured over a 10 mm length centered on the stenotic lesion. A higher value indicates more significant inflammatory infiltration of local adipose tissue. For patients with multiple stenoses, the most severe lesion was selected, and its change was dynamically tracked (△FAI = post-treatment − pre-treatment).
(c) WSS Assessment: WSS reflects the frictional force exerted by blood flow on the vascular endothelium. WSS values were extracted at the most severe stenotic lesion. The change before and after intervention was tracked (△WSS = post-treatment − pre-treatment).
(d) High-Risk Plaque Characterization: Based on the CAD-RADS™ 2.0 criteria (2), morphological instability features of the plaques were identified. A plaque must exhibit two or more of the following features to be classified as a high-risk plaque: ① Spotty calcification: Small, punctate calcified deposits observed within the plaque; ② Low-attenuation plaque: Non-calcified plaque areas with internal CT attenuation values below 30 HU; ③ Positive remodeling: The ratio of the outer vessel diameter at the plaque site to the average of the normal proximal and distal reference diameters is >1.1; ④ Napkin-ring sign: The simultaneous presence of two features on the cross-sectional image of a non-calcified plaque: a central low-attenuation core surrounded by a ring-like peripheral rim with higher CT attenuation.

### 2.7 Sample size

There are no studies using coronary artery hemodynamic parameters and FAI to evaluate the efficacy of traditional Chinese medicine in treating SCAD. This study is an exploratory trial and does not require sample size calculations (44). Sixty subjects will be recruited, with 30 participants allocated to each group, to investigate our research objectives.

### 2.8 Data collection and management

This study utilizes paper-based CRFs for data collection. Physical examinations, scale scores, and results of tests and inspections shall be recorded on the CRFs promptly, accurately, completely, and in a standardized manner. Clinical monitors from the Clinical Pharmacology Research Institute of Xiyuan Hospital will review the data for completeness annually, after which the data will be transferred to two researchers for independent, double data entry to ensure accuracy. All data will be securely stored in a password-protected database, accessible only to the principal investigator for analysis and reporting. Other researchers will not have access to these data. The paper CRF forms and signed consent forms will be stored in a locked filing cabinet at Xiyuan Hospital. After all participants complete follow-up, all data will be stored electronically in the database of Xiyuan Hospital. participants' paper documents will be stored in a locked filing cabinet at Xiyuan Hospital for five years.

### 2.9 Statistical analysis

According to the ITT analysis principle, the Full Analysis Set (FAS) is defined as the set of qualified cases that completed the study and analyzable drop-out cases, excluding excluded cases. The Per Protocol Set (PPS) is defined as the set of cases that comply with the trial protocol, have not concurrently used other drugs or treatments that may affect the trial results, have completed the full intervention and follow-up, and have completed the CRF as required. The Safe Set (SS) is defined as the set of cases that have received at least one dose of medication, possess baseline safety data, and have undergone at least one safety visit. Efficacy and safety will be evaluated based on the FAS, with the PPS used for sensitivity analysis.

Continuous variables will be described as mean ± standard deviation, categorical variables as frequency and percentage. Comparison of outcome measures between groups: continuous variables with a normal distribution will be analyzed using paired *t*-tests; those without a normal distribution will be analyzed using the Wilcoxon rank-sum test. Categorical variables will be compared using the *χ*^2^ test or Fisher's exact test. Subgroup analyses will be conducted based on whether patients have high-risk plaques (positive remodeling, low-attenuation plaque, spotty calcification, and napkin-ring sign) (2). Additionally, difficult-to-control and intergroup imbalanced confounding factors will be analyzed as covariates using analysis of covariance to eliminate their impact on efficacy evaluation. *P*-value <0.05 will be considered statistically significant. All statistical analyses will be performed using SPSS (version 25).

## 3 Discussion

The treatment paradigm for SCAD is currently undergoing a profound transformation: shifting from a primary focus on ischemia relief toward plaque stabilization; from reliance on invasive coronary imaging to the increasing application of CCTA for non-invasive coronary assessment; and from single-target lipid-lowering therapy to a more comprehensive approach addressing residual metabolic, inflammatory, and thrombotic risks (45, 46). Despite receiving GDMT, patients with SCAD and moderate stenosis continue to exhibit suboptimal outcomes. The ORFAN study indicates that the incidence of MACE events in this population is nearly twice that of patients with severe stenosis (3). Similarly, the PROMISE study reported that patients with coronary or branch stenosis <70% experienced higher event rates compared to those with severe stenosis (4). Colchicine has been recommended by leading international cardiology guidelines as an adjunctive therapy to reduce cardiovascular risk and mitigate residual inflammation (15, 47). However, recent evidence from the CLEAR SYNERGY study has cast doubt on these conclusions (14). A meta-analysis by Boracchi et al. demonstrated that colchicine reduced the risk of myocardial infarction by 34% in patients with SCAD, but did not improve prognosis (48). These findings highlight the need to explore novel therapeutic strategies. Investigating the effects of Traditional Chinese medicine (TCM) on symptoms, plaque stabilization, and clinical outcomes in SCAD with moderate stenosis may provide new avenues for optimizing treatment.

Academician Ge Junbo highlighted that coronary plaque and inflammation are stronger cardiovascular event predictors than LDL-C, emphasizing the critical value of FAI in assessing residual inflammatory risk (17). Coronary hemodynamic indices also provide valuable insights for risk prediction. CT-FFR enables more precise evaluation of moderate stenoses, reducing overdiagnosis and unnecessary interventions (49). Moreover, low WSS has been implicated in the initiation and progression of atherosclerotic plaques and in promoting plaque rupture (50). Consequently, CCTA-guided preventive management of patients with SCAD and moderate stenosis offers significant clinical benefit by reducing cardiac death and myocardial infarction, minimizing unnecessary revascularization, and alleviating overall disease burden.

TCM provides a unique therapeutic modality that acts through multiple targets and pathways. The integration of Chinese and Western medicine is increasingly regarded as a promising strategy for cardiovascular intervention (29, 51). However, previous studies investigating TCM for SCAD have rarely employed objective imaging-based endpoints such as CCTA, and their treatment durations were generally limited, restricting the ability to assess long-term effects. This study implemented a 6-month intervention period and combined advanced imaging techniques—including FAI and hemodynamic assessments—to evaluate the comprehensive effects of SYF on SCAD in a systematic and evidence-based manner. Furthermore, regular safety monitoring was incorporated to dynamically assess bleeding risk and overall drug safety, thereby generating robust evidence to support clinical application.

Several limitations of this study warrant consideration. First, due to the intrinsic characteristics of CCTA, its soft tissue resolution remains inferior to intravascular ultrasound and is influenced by heart rate and respiratory motion, which may affect the accuracy of plaque characterization. To mitigate these limitations, we employed the GE Revolution CT scanner, an advanced system with enhanced tolerance to heart rate variability and spontaneous respiration, as well as a wide-body detector that enables rapid scanning, improved spatial resolution, and artifact reduction. In addition, all CCTA images were interpreted by experienced radiologists to optimize assessment reliability. Second, this study did not incorporate long-term post-treatment follow-up, limiting our ability to evaluate the impact of SYF on long-term prognosis. To address this, we plan to maintain follow-up with participants beyond the study period to monitor adverse events and further elucidate the sustained effects of SYF in SCAD with moderate stenosis.
