# Supplementary material for: Efficacy and safety of Shenqi Yangxin formula in patients with stable coronary artery disease based on CCTA: rationale, design, and study protocol for a randomized, double-blind, placebo-controlled study
Source: Front Cardiovasc Med. 2026 May 8;13:1697750. doi: 10.3389/fcvm.2026.1697750 (PMC13193870; doi:10.3389/fcvm.2026.1697750)
Supplement: Supplementary file 1 [file Datasheet1.pdf]

frontiers

Supplementary File 1

UPLC-Q-TOF-MS of SYF

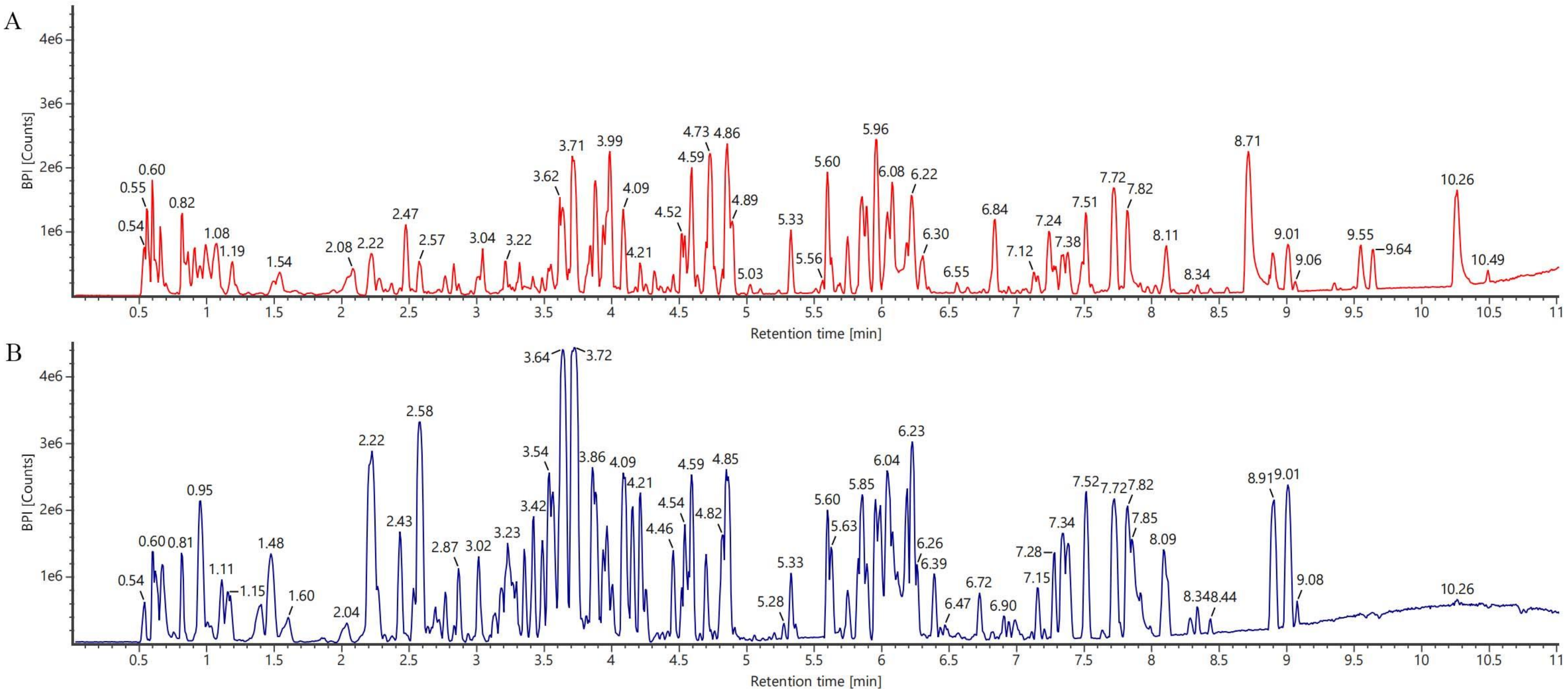

Fig.1. UPLC-Q-TOF-MS-based chemical fingerprint of SYF. (A) Total ion chromatogram of SYF acquired in the positive ion mode. (B) Total ion chromatogram of SYF acquired in the negative ion mode.

Supplementary File 2

Chemical constituents of SYF

No.	Component name	Formula	Observed （m/z）	Observed RT (min)	Adducts	Mass error (ppm)	Herbal source
1	Chikusetsusaponin IVa	$C_{42}H_{66}O_{14}$	793.44	7.34	[M-H]-	-0.10	Cyathulae Radix，Ginseng Radix et Rhizoma
2	Chuanxiongol	$C_{13}H_{14}O_3$	219.102	4.10	[M+H]+	5.30	Chuanxiong Rhizoma
3	Ginsenoside Rg4	$C_{42}H_{70}O_{12}$	811.48	7.28	[M+FA-H]-	0.50	Ginseng Radix et Rhizoma
4	Ginsenoside Rg5	$C_{42}H_{70}O_{12}$	811.48	8.90	[M+FA-H]-	0.80	Ginseng Radix et Rhizoma
5	Notoginsenoside R2	$C_{42}H_{72}O_{13}$	815.48	5.91	[M+FA-H]-	-1.00	Ginseng Radix et Rhizoma, Notoginseng Radix
6	Notoginsenoside T5	$C_{41}H_{68}O_{12}$	797.47	7.21	[M+FA-H]-	-0.70	Notoginseng Radix
7	Octahydrocurcumin	$C_{21}H_{28}O_6$	399.18	9.55	[M+Na]+	1.70	Curcumae Radix
8	senkyunolide B	$C_{12}H_{12}O_3$	205.09	7.83	[M+H]+	4.10	Chuanxiong Rhizoma
9	Senkyunolide D	$C_{12}H_{14}O_4$	223.10	3.88	[M+H]+	-0.10	Chuanxiong Rhizoma
10	Vanillic acid	$C_8H_8O_4$	167.03	3.14	[M-H]-	0.80	Forsythiae Fructus, Ginseng Radix et Rhizoma, Chuanxiong Rhizoma, Curcumae Radix
11	Verbascoside	$C_{29}H_{36}O_{15}$	625.21	3.64	[M+H]+	1.70	Forsythiae Fructus
12	25R Inokosterone	$C_{27}H_{44}O_7$	525.31	3.96	[M+FA-H]-	1.60	Cyathulae Radix

Supplementary File 3

SPIRIT 2013 Checklist

**Section/item**	**Item No**	**Description**	**Addressed on section headings**
Administrative information			
Title	1	Descriptive title identifying the study design, population, interventions, and, if applicable, trial acronym	Title page
Trial registration	2a	Trial identifier and registry name. If not yet registered, name of intended registry	Trial registration number
	2b	All items from the World Health Organization Trial Registration Data Set	2.1 Study design
Protocol version	3	Date and version identifier	2.1 Study design
Funding	4	Sources and types of financial, material, and other support	Funding
Roles and responsibilities	5a	Names, affiliations, and roles of protocol contributors	Title page
	5b	Name and contact information for the trial sponsor	Funding
	5c	Role of study sponsor and funders, if any, in study design; collection, management, analysis, and interpretation of data; writing of the report; and the decision to submit the report for publication, including whether they will have ultimate authority over any of these activities	Author Contributions, Funding
	5d	Composition, roles, and responsibilities of the coordinating centre, steering committee, endpoint adjudication committee, data management team, and other individuals or groups overseeing the trial, if applicable (see Item 21a for data monitoring committee)	2.8 Data collection and management
Introduction			

Background and rationale	6a	Description of research question and justification for undertaking the trial, including summary of relevant studies (published and unpublished) examining benefits and harms for each intervention	Background
	6b	Explanation for choice of comparators	2.5.2 Control group
Objectives	7	Specific objectives or hypotheses	Introduction
Trial design	8	Description of trial design including type of trial (eg, parallel group, crossover, factorial, single group), allocation ratio, and framework (eg, superiority, equivalence, noninferiority, exploratory)	Methods
Methods: Participants, interventions, and outcomes			
Study setting	9	Description of study settings (eg, community clinic, academic hospital) and list of countries where data will be collected. Reference to where list of study sites can be obtained	2.1 Study design
Eligibility criteria	10	Inclusion and exclusion criteria for participants. If applicable, eligibility criteria for study centres and individuals who will perform the interventions (eg, surgeons, psychotherapists)	2.2 Participants
Interventions	11a	Interventions for each group with sufficient detail to allow replication, including how and when they will be administered	2.5 Interventions
	11b	Criteria for discontinuing or modifying allocated interventions for a given trial participant (eg, drug dose change in response to harms, participant request, or improving/worsening disease)	2.5.3 Removal, dropout, and termination criteria
	11c	Strategies to improve adherence to intervention protocols, and any procedures for monitoring adherence (eg, drug tablet return, laboratory tests)	Methods
	11d	Relevant concomitant care and interventions that are permitted or prohibited during the trial	2.5.4 Drugs combined and contraindicated in trial
Outcomes	12	Primary, secondary, and other outcomes, including the specific measurement variable (eg, systolic blood pressure), analysis metric (eg, change from baseline, final value, time to event), method of aggregation (eg,	2.6 Outcomes

		median, proportion), and time point for each outcome. Explanation of the clinical relevance of chosen efficacy and harm outcomes is strongly recommended	
Participant timeline	13	Time schedule of enrolment, interventions (including any run-ins and washouts), assessments, and visits for participants. A schematic diagram is highly recommended (see Figure)	Fig.1, Table 1
Sample size	14	Estimated number of participants needed to achieve study objectives and how it was determined, including clinical and statistical assumptions supporting any sample size calculations	2.7 Sample size
Recruitment	15	Strategies for achieving adequate participant enrolment to reach target sample size	2.2.1 Recruitment
Methods: Assignment of interventions (for controlled trials)			
Allocation:			
Sequence generation	16a	Method of generating the allocation sequence (eg, computer-generated random numbers), and list of any factors for stratification. To reduce predictability of a random sequence, details of any planned restriction (eg, blocking) should be provided in a separate document that is unavailable to those who enrol participants or assign interventions	2.3 Randomization and allocation concealment
Allocation concealment mechanism	16b	Mechanism of implementing the allocation sequence (eg, central telephone; sequentially numbered, opaque, sealed envelopes), describing any steps to conceal the sequence until interventions are assigned	2.3 Randomization and allocation concealment
Implementation	16c	Who will generate the allocation sequence, who will enrol participants, and who will assign participants to interventions	2.3 Randomization and allocation concealment
Blinding (masking)	17a	Who will be blinded after assignment to interventions (eg, trial participants, care providers, outcome assessors, data analysts), and how	2.4 Blinding
	17b	If blinded, circumstances under which unblinding is permissible, and procedure for revealing a participant's allocated intervention during the trial	2.4 Blinding
Methods: Data collection, management, and analysis			
Data collection	18a	Plans for assessment and collection of outcome, baseline, and other trial data, including any related	2.6 Outcomes

methods		processes to promote data quality (eg, duplicate measurements, training of assessors) and a description of study instruments (eg, questionnaires, laboratory tests) along with their reliability and validity, if known. Reference to where data collection forms can be found, if not in the protocol	
	18b	Plans to promote participant retention and complete follow-up, including list of any outcome data to be collected for participants who discontinue or deviate from intervention protocols	2.5.3 Removal, dropout, and termination criteria
Data management	19	Plans for data entry, coding, security, and storage, including any related processes to promote data quality (eg, double data entry; range checks for data values). Reference to where details of data management procedures can be found, if not in the protocol	2.8 Data collection and management
Statistical methods	20a	Statistical methods for analysing primary and secondary outcomes. Reference to where other details of the statistical analysis plan can be found, if not in the protocol	2.9 Statistical analysis
	20b	Methods for any additional analyses (eg, subgroup and adjusted analyses)	2.9 Statistical analysis
	20c	Definition of analysis population relating to protocol non-adherence (eg, as randomised analysis), and any statistical methods to handle missing data (eg, multiple imputation)	2.9 Statistical analysis
Methods: Monitoring			
Data monitoring	21a	Composition of data monitoring committee (DMC); summary of its role and reporting structure; statement of whether it is independent from the sponsor and competing interests; and reference to where further details about its charter can be found, if not in the protocol. Alternatively, an explanation of why a DMC is not needed	2.8 Data collection and management
	21b	Description of any interim analyses and stopping guidelines, including who will have access to these interim results and make the final decision to terminate the trial	2.5.3 Removal, dropout, and termination criteria
Harms	22	Plans for collecting, assessing, reporting, and managing solicited and spontaneously reported adverse events and other unintended effects of trial interventions or trial conduct	2.6 Outcomes
Auditing	23	Frequency and procedures for auditing trial conduct, if any, and whether the process will be independent	2.8 Data collection

		from investigators and the sponsor	and management
Ethics and dissemination			
Research ethics approval	24	Plans for seeking research ethics committee/institutional review board (REC/IRB) approval	2.1 Study design
Protocol amendments	25	Plans for communicating important protocol modifications (eg, changes to eligibility criteria, outcomes, analyses) to relevant parties (eg, investigators, REC/IRBs, trial participants, trial registries, journals, regulators)	2.1 Study design
Consent or assent	26a	Who will obtain informed consent or assent from potential trial participants or authorised surrogates, and how (see Item 32)	2.2 Participants
	26b	Additional consent provisions for collection and use of participant data and biological specimens in ancillary studies, if applicable	N/A
Confidentiality	27	How personal information about potential and enrolled participants will be collected, shared, and maintained in order to protect confidentiality before, during, and after the trial	2.8 Data collection and management
Declaration of interests	28	Financial and other competing interests for principal investigators for the overall trial and each study site	Conflict of Interest
Access to data	29	Statement of who will have access to the final trial dataset, and disclosure of contractual agreements that limit such access for investigators	2.8 Data collection and management
Ancillary and post-trial care	30	Provisions, if any, for ancillary and post-trial care, and for compensation to those who suffer harm from trial participation	N/A
Dissemination policy	31a	Plans for investigators and sponsor to communicate trial results to participants, healthcare professionals, the public, and other relevant groups (eg, via publication, reporting in results databases, or other data sharing arrangements), including any publication restrictions	N/A
	31b	Authorship eligibility guidelines and any intended use of professional writers	N/A
	31c	Plans, if any, for granting public access to the full protocol, participant-level dataset, and statistical code	N/A
Appendices			

Informed consent materials	32	Model consent form and other related documentation given to participants and authorised surrogates	Supplementary file 4
Biological specimens	33	Plans for collection, laboratory evaluation, and storage of biological specimens for genetic or molecular analysis in the current trial and for future use in ancillary studies, if applicable	2.6 Outcomes

*It is strongly recommended that this checklist be read in conjunction with the SPIRIT 2013 Explanation & Elaboration for important clarification on the items. Amendments to the protocol should be tracked and dated. The SPIRIT checklist is copyrighted by the SPIRIT Group under the Creative Commons "Attribution-NonCommercial-NoDerivs 3.0 Unported" license.

Supplementary File 4

Informed Consent Form

Dear Patient: Greetings!

Your physician has diagnosed you with stable coronary heart disease (SCAD, Qi deficiency and blood stasis syndrome). We sincerely welcome your voluntary participation in the clinical trial of Shenqi Yangxin Formula and express our heartfelt gratitude for your involvement. Before deciding whether to participate, it is important that you understand the purpose of this trial, the investigational medication, the potential risks involved, the expectations during the trial, and your rights as a participant. Please read this participant information sheet carefully.

1. Background

Currently, the treatment and management of SCAD are undergoing a historic transformation. It is recognized that patients with moderate coronary artery stenosis also carry a high risk of adverse cardiovascular events. Invasive revascularization therapy does not improve the prognosis of SCAD, and reducing residual cardiovascular risk may be an effective approach to further enhance patient outcomes. Based on coronary computed tomography angiography (CCTA) derived technology, evaluating plaque burden and hemodynamics in patients with SCAD, as well as performing risk stratification and prediction, has become a hot topic in current research and is of great significance for standardizing the clinical management of patients with SCAD.

Shenqi Yangxin Formula (SYF) is derived from Professor Meizhong Yue's effective prescription for treating Coronary Heart Disease, has been used in China for many years to treat patients with SCAD and Qi deficiency and blood stasis syndrome. IT has been shown to improve clinical symptoms and adverse reactions are generally rare. However, the mechanism of benefit remains unclear. Based on this, we designed this trial to evaluate the efficacy and safety of SYF in the treatment of SCAD.

2.Study design

The purpose of this study is to objectively evaluate the efficacy and safety of SYF in improving SCAD by assessing CCTA-derived indexes, including coronary hemodynamic parameters, FAI, imaging characteristics of plaques, etc.

This study is a prospective, double-blind, randomized controlled trial to be conducted at Xiyuan Hospital, with an anticipated enrollment of 60 voluntary participants, including 30 in the experimental group and 30 in the control group. Participants will be assigned to the experimental or control group with equal probability.

This study has been approved by Xiyuan Hospital of the China Academy of Chinese Medical Sciences. The Ethics Committee of Xiyuan Hospital of the China Academy of Chinese Medical Sciences has reviewed this study and confirmed that it complies with the principles of the Declaration of Helsinki and adheres to medical ethics.

3.Eligibility Criteria for Participation

a. CCTA demonstrates at least one major branch with luminal diameter stenosis of 50% -70%, exhibiting soft plaque or mixed plaque types.

b. Based on CCTA analysis within the past month, CT-FFR > 0.8;

c. Canadian Cardiovascular Society classification of angina pectoris is grade I -II.

d. Meets the diagnostic criteria for Qi deficiency and blood stasis syndrome;

e. Aged 30 -75 years, with no gender restrictions;

f. Voluntarily consents to participate and signs the informed consent form.

4. Exclusion Criteria for Participation

a. Acute myocardial infarction or coronary revascularization treatment within the past 30 days.

b. Presence of any of the following conditions: left main coronary artery stenosis ≥50% or chronic total occlusion; drug-resistant hypertension (systolic blood pressure ≥160 mmHg, diastolic blood pressure ≥ 100 mmHg); severe malignant arrhythmia, cor pulmonale, rheumatic heart disease, myocarditis, cardiomyopathy, aortic dissection, pulmonary embolism, or other serious diseases.

c. Patients who have undergone coronary artery bypass graft surgery；

d. Patients with stents in both the left and right coronary arteries；

e. Patients presenting with chest pain symptoms related to cervical spondylosis, biliary heart syndrome, esophageal hiatal hernia, neurasthenia, menopausal syndrome, or other related conditions；

f. Patients with congenital coronary artery anomalies or connective tissue disease with coronary artery involvement;

g. Individuals at high risk of severe bleeding；

h. Patients with severe liver disease, or alanine aminotransferase (ALT) and aspartate aminotransferase (AST) levels exceeding three times the upper limit of normal, or those with renal impairment, defined as creatinine clearance < 60 ml/min；

i. Patients with severe hematological disorders or malignant tumors；

j. Individuals with any of the following contraindications to coronary CTA: ① Patients with contraindications to Ioversol injection (e.g., a history of severe hypersensitivity reactions, severe thyroid disease, renal insufficiency, or any other conditions deemed inappropriate for contrast administration by the investigators). ② Inability to cooperate with scanning and breath-holding. ③ Pregnant or lactating women, or women of childbearing potential planning pregnancy. ④ Clinically unstable vital signs (such as decompensated heart failure, severe hypotension, etc.)；

k. Patients with psychiatric disorders or cognitive impairment；

l. Patients allergic to the investigational drug；

m. Participation in other clinical trials within the past month, or oral administration of other Chinese herbal preparations with blood-activating and stasis-resolving effects；

n. Patients whose CCTA images are unavailable or of insufficient quality to permit plaque characterization or hemodynamic analysis

5. Overall Procedure

At the start of the study, an informed consent form will be obtained from participants who preliminarily meet the inclusion criteria. Medical history and basic information will be collected. General clinical data will be collected, including body temperature, pulse, respiration, blood pressure, body weight, height, tongue appearance, and pulse condition. CCTA, echocardiographic, 12-lead electrocardiogram, complete blood count, liver and kidney function tests, coagulation function, platelet aggregation rate, blood glucose and lipid profiles, as well as urine and fecal routine examinations, and untargeted metabolomics analysis. Evaluate Traditional Chinese Medicine syndrome score(TCMSS), Seattle Angina Questionnaire scores (SAQ). Provide health education for patients with coronary atherosclerotic heart disease.

Patients in the experimental group will receive SYF combined with GDMT, while patients in the control group will receive placebo combined with GDMT, with a total treatment duration of 24 weeks. SYF includes Shenqi Yangxin granules (SYG), packaged as 9.65g per bag, and Notoginseng Radix powder (NRP), packaged in 3 g per bag. During the first 12 weeks, take one bag of SYG and half a bag of NRP per dose, twice daily. During the second 12 weeks, take one bag of SYG and half a bag of NRP per dose, once daily. The placebo containing 10% of the original medicine, was administered in the same manner.

At weeks 4 and 12 of the study, you need to visit the hospital and truthfully report any changes in your condition to the physician, who will collect your symptoms and physical examination results, TCMSS and SAQ. Blood samples will be collected for blood routine examination, liver and kidney function tests, coagulation function, platelet aggregation rate, blood glucose and lipid profiles. Routine urine and stool examinations will be performed. 12-lead Electrocardiogram will be conducted.

At week 24 of the study: The study concludes at this time. You should visit the hospital. The physician will inquire and record the changes in your condition and conduct a comprehensive physical examination, including body temperature, pulse, blood pressure, respiration, etc. Assessment of TCMSS and SAQ. Undergo CCTA, echocardiography, 12-lead electrocardiogram. Blood will be drawn for blood routine examination, liver and kidney function tests, coagulation function, platelet aggregation rate, blood glucose and lipid profiles. 5 ml of blood will be collected and stored in our hospital’s biobank for untargeted metabolomics analysis. Routine urinalysis and stool examination samples will also be collected.

Please attend follow-up visits according to the schedule agreed upon with your physician. Your follow-up is very important, as the physician will assess whether the treatment you received is truly effective.

Please take the medication as directed by your physician, and promptly and objectively record your condition after each dose. At the same time, please record any medications you must continue to take for other conditions during the clinical trial.

During the clinical trial, except for the medications specified in the protocol, please do not use other traditional Chinese medicines, proprietary Chinese medicines, or related non-pharmacological TCM therapies for treating SCAD. If you require other treatments, please consult your physician in advance.

6.Your Rights and Interests

a. The investigators will explain the investigational drug and trial procedures to you. Participation is entirely voluntary. The investigator will inform you of all events related to you, allowing you to decide at any time whether to continue participation. If you have any questions, you may call or consult the investigator directly. You will receive appropriate medical care throughout the study. You may refuse to participate in this study or withdraw at any time during the study. This will not affect your relationship with your physician, nor will it affect your medical care or any other benefits. You may withdraw from this study at any time without discrimination or unfair treatment, and your medical care and rights will not be affected. You are not required to participate in this study in order to receive treatment for your illness. If you withdraw from the study due to medication-related reasons, informing the physician of any changes in our condition and completing the corresponding physical and physicochemical examinations will be highly beneficial for both your health and the overall study.
b. You and society may potentially benefit from this clinical trial. These benefits include possible improvement in your condition, and your participation will also contribute to the development of a new medication for treating this disease. This study may help develop a new treatment method for other patients with similar conditions.

7.Regarding Costs

In this study, SYF, placebo and examination items (include CCTA 1 time, echocardiography 1 time, routine blood examination 3 times, liver and kidney function 3 times, coagulation function 3 times, platelet aggregation rate 3 times, rapid blood glucose test 3 times, blood lipid levels 3 times, 12-lead electrocardiogram 3 times, untargeted metabolomics 1 time, routine urinalysis and stool examination 3 times) will be covered by the clinical trial. Your GDMT for SCAD and as well as treatments and examinations required for other comorbidities, will not be covered free of charge.

8.Your Obligations

Adhere to the principle of voluntary participation and sign the informed consent form before the clinical trial commences.

Follow the clinical trial protocol, comply with the investigator’s unified arrangements, and cooperate with the investigator to complete the trial tasks.

During the trial, take medication as directed by the physician. At each follow-up visit, unused medication and its packaging must be returned. If other medications are required, please contact the investigator.

9. Possible Adverse Reactions and Safety Measures

All therapeutic drugs may cause side effects. In this study, the possible adverse reactions associated with the use of SYF include mild diarrhea， which usually resolves spontaneously after discontinuing the medication. If any harm or illness related to the study occurs, or if adverse reactions arise from the use of SYF, you should immediately contact your physician, who will provide necessary diagnosis, treatment, and advice.

The physician will make every effort to prevent and treat any injuries that may result from this study. In the event of study-related harm, Xiyuan Hospital shall be responsible for compensation. If adverse events such as angina pectoris occur during medication, the following measures should be taken: 1) Immediately stop all activities, including physical labor and emotionally agitating actions; find a safe place to sit or lean while standing to avoid increasing cardiac workload. 2) Place nitroglycerin under the tongue or take fast-acting heart medicine, etc.. 3) Maintain deep respiration and avoid tension and panic. 4) If accompanied by dyspnea, assume a semi-recumbent or sitting position with legs hanging down to reduce cardiac load and improve respiration. 5) If conditions permit, oxygen therapy may be administered to help improve myocardial hypoxia. 6) In the event of cardiac arrest, correct manual cardiopulmonary resuscitation should be performed immediately, and professional medical personnel should be contacted as soon as possible. 7) After an angina attack, even if symptoms have subsided, you should promptly go to the hospital for further diagnosis and treatment to prevent deterioration or acute myocardial infarction.

Any damages caused by GDMT for SCAD or other treatments for diseases during the study period are not covered by compensation.

During the study period, you are required to attend hospital follow-up visits on schedule and undergo examinations, which may cause you inconvenience or discomfort.

10. Reasons for Termination of Your Participation in the Clinical Trial

a . The investigator determines that continuing the trial would be harmful to you.

b. You experience serious adverse events or special physiological changes, including cardiac death, non-fatal myocardial infarction, coronary revascularization, stroke, severe angina pectoris, hospitalization due to acute coronary syndrome, malignant arrhythmia, heart failure, or other bleeding/thrombotic complications.

c. Due to ineffective treatment or progression of the disease caused by comorbid conditions or other reasons.

d. You did not take the medication as instructed by the physician.

e. Serious adverse drug reactions occurred during the study.

If any of the above situations occur, the investigator has the right to terminate your participation in the clinical trial without your consent.

11. Confidentiality

All information related to you, including your identity, medical history, condition, physical examination, and laboratory test results, will be strictly kept confidential within the limits permitted by law. Only authorized investigators, the ethics committee, and the research funding department may access your original medical records to verify the authenticity and accuracy of the data collected in this study, without involving your personal private information. Your name will not appear in any publicly available materials or reports related to this study.

12. Publication

Regardless of the research results, we will make every effort to publish them.

Thanks for reading the above information. If you decide to participate in the clinical trial, please inform your physician, who will arrange all matters related to the clinical trial for you.

Please keep this information.
